# Intrinsic Cell-Class–Specific Modulation of Intracellular Chloride Levels and Inhibitory Function, in Cortical Networks, between Day and Night

**DOI:** 10.1523/ENEURO.0325-25.2025

**Published:** 2025-12-18

**Authors:** Laura Alberio, Amy Marshall, Robert T. Graham, Connie MacKenzie-Gray Scott, Luciano Saieva, Sarah E. Gartside, Gian Michele Ratto, Andrew J. Trevelyan

**Affiliations:** ^1^Faculty of Medical Science, Biosciences Institute, Newcastle University, Newcastle upon Tyne NE2 4HH, United Kingdom; ^2^Scuola Normale Superiore, Istituto Nanoscienze CNR and NEST, Pisa 56127, Italy

**Keywords:** chloride, interneuron, neocortex, parvalbumin, pyramidal cell, somatostatin

## Abstract

Recent work showed unexpectedly large, daily modulation of intracellular chloride concentration ([Cl^−^]_in_) in cortical pyramidal cells, with consequences for GABAergic function and network excitability ( Alfonsa et al., 2023; Pracucci et al., 2023). One explanation for this [Cl^−^]_in_ modulation is that it arises from variation in presynaptic drive. In that case, neuronal classes with similar synaptic inputs should show correlated changes in activity-dependent ionic redistribution. To examine this prediction, we performed in vivo, LSSm-ClopHensor imaging to measure [Cl^−^]_in_ and pH_in_ in populations of parvalbumin- (PV) and somatostatin (SST)-expressing interneurons in neocortical Layer 2/3 of male and female adult mice. Imaging was performed at zeitgeber time (ZT)5 and ZT17, when pyramidal cell [Cl^−^]_in_ shows maximal divergence ( Pracucci et al., 2023). Interestingly, PV interneurons also showed large physiological [Cl^−^]_in_ modulation between these times but out-of-phase with that in pyramidal cells, being raised at ZT5 and lower at ZT17, and with a far higher mean [Cl^−^]_in_. SST interneurons showed less modulation, with higher variance, and with a temporal dynamic resembling the pyramidal cell pattern. Notably, in vitro experimental assays of inhibition, involving these two classes of interneuron, differed markedly at ZT5 and ZT17. The persistence of these time-of-day effects in vitro and the difference in [Cl^−^]_in_ dynamics between pyramidal cells and PV interneurons in vivo both point toward cell-intrinsic regulation being more important than activity-dependent effects in setting these slow, daily, physiological, ionic redistribution patterns. We discuss what other possible factors may influence variations in brain state through the day.

## Significance Statement

We find that the three largest subclasses of supragranular neocortical neurons, pyramidal cells and parvalbumin- (PV) and somatostatin-expressing interneurons, show different patterns of daily modulation of [Cl^−^]_in_. Notably, the modulation in PV interneurons is out-of-phase with the other two cell classes. We further observed differences in network inhibition in brain slices prepared at ZT5 and ZT17. We argue, based upon these various lines of evidence, that activity-dependent ionic redistribution is not the primary determinant of the slow daily [Cl^−^]_in_ modulation. Instead, we discuss which cell-autonomous mechanisms may be involved and what implications these findings have for our understanding of brain state differences.

## Introduction

Cortical activity patterns vary markedly throughout the day (Vyazovskiy et al., 2009; Watson et al., 2016; Torrado Pacheco et al., 2021), as the brain cycles through different brain states. However, the physiological basis of this variable activity is poorly understood. Clinical studies also observe daily fluctuations in neurological and psychiatric symptoms (Murray and Harvey, 2010; Wulff et al., 2010; Karoly et al., 2016, 2017, 2021; Logan and McClung, 2019). These findings likely relate to the many documented changes in cellular function from day to night, including the number, structure, strength, and plasticity of synapses (Tononi and Cirelli, 2006; Vyazovskiy et al., 2008; Liu et al., 2010; Snider et al., 2018; Hartsock and Spencer, 2020; Zhou et al., 2020), metabolic state (Natsubori et al., 2020), and gene expression and protein phosphorylation (Wang et al., 2018; Bruning et al., 2019).

Another influence on brain activity, unrecognized until recently, is the fluctuating pattern of ionic redistribution between brain compartments (Ding et al., 2016; Alfonsa et al., 2023; Pracucci et al., 2023; Untiet et al., 2023). Of special note are two studies showing that intracellular chloride levels ([Cl^−^]_in_), in mouse neocortical pyramidal cells, differ markedly between day [zeitgeber time (ZT)5] and night (ZT17; 12 h light/12 h dark regime; ZT0 is when lights are switched on; Alfonsa et al., 2023; Pracucci et al., 2023). Using the chloride biosensor, LSSm-ClopHensor (Arosio et al., 2010; Sulis Sato et al., 2017; Lodovichi et al., 2021), to examine large populations of pyramidal cells, we estimated that [Cl^−^]_in_ approximately doubles from ZT5, when mice are resting, to ZT17, when they are active (Pracucci et al., 2023). Furthermore, the increase in [Cl^−^]_in_ was associated with a marked increase in network excitability (Pracucci et al., 2023). Given that GABAergic activity causes inward movement of Cl^−^ ions (Thompson and Gahwiler, 1989; Staley et al., 1995; Kaila et al., 1997; Viitanen et al., 2010), an effect that is exacerbated when GABAergic and glutamatergic drives coincide (Trevelyan, 2009), one explanation for the increased [Cl^−^]_in_ at night is that this simply reflects a raised level of network activity.

Alternatively, [Cl^−^]_in_ dynamics may be more influenced by intrinsic cellular mechanisms; for instance, in response to changes in molecular crowding leading to compensatory ionic redistribution, as has been described in multiple different cell classes (Stangherlin et al., 2021) although notably not yet in neurons. The crowding changes, caused by temporal segregation of protein translation and degradation to opposite sides of the circadian cycle, lead to changes in functional levels of the cation–chloride cotransporters, KCC2 and NKCC1 (Rivera et al., 1999; Pracucci et al., 2023), possibly through the crowding sensing capacity of WNK (Boyd-Shiwarski et al., 2022).

If extrinsic network activity is the primary determinant of chloride redistribution, then neurons that receive similar presynaptic drive should show correlated [Cl^−^]_in_ dynamics. We therefore examined whether other classes of cortical neuron show similar [Cl^−^]_in_ dynamics to those found in pyramidal cells. To address this, we performed in vivo imaging of mice expressing LSSm-ClopHensor under either the parvalbumin (PV) or somatostatin (SST) promoters, in the same cortical territory, in the supragranular neocortex of the dorsal somatosensory area.

Notably, Alfonsa et al. (2023) found differences in [Cl^−^]_in_ in brain slices prepared either at ZT3 or ZT15, using gramicidin perforated-patch–clamp recordings, even though brain slices are typically very quiescent. We therefore examined whether mouse brain slices prepared at different times of day also vary in assays of neocortical inhibitory function. First, we assayed the pattern of feedforward inhibition in response to a train of excitatory drive, which involves both PV and SST interneurons (Pouille and Scanziani, 2004); next, we recorded a gradually increasing optogenetic excitatory drive to the adjacent cortical area, which induces a prominent γ-oscillation (Adesnik et al., 2012). We discuss different explanations for these time-of-day effects on cortical function.

## Materials and Methods

All reagents were purchased from Sigma/Merck, unless otherwise stated.

### Animals and housing conditions

All animal handling and experimentations were performed according to the guidelines of the UK Home Office and Animals (Scientific Procedures) Act of 1986 and approved by the Newcastle University Animal Welfare and Ethical Review Body.

Mice were housed on a 12/12 h light/dark cycle with *ad libitum* access to food and water. Chloride imaging and immunolabeling were performed on adult (>2 months old) C57Bl/6 male and female mice purchased from Jackson Laboratory: mouse lines 008069 (PV-Cre), and 018973 (SST-Cre). The imaging data presented in this paper have been obtained from a total of 11 SST mice (four males and seven females) and 10 PV mice (eight males and two females). The in vitro studies were performed on 10 mice, 6 of which were killed at ZT5 (four males, two females, 17 brain slices) and 4 of which were killed at ZT17 (three males, one female, 16 brain slices). No sex differences were observed, and so all data were pooled within the experimental groupings. The pyramidal [Cl^−^]_in_ and pH_in_ have been reported previously (Pracucci et al., 2023) and are included here solely for comparison with the interneuron measurements. Pyramidal clopHensor expression was achieved using the same floxed viral vectors, injected into Emx1 Cre-recombinase mice (Jackson Laboratory line #5628); full details of that dataset are found within this prior publication (Pracucci et al., 2023).

### Production and purification of the AAV8-EF1a-DIO-ClopHensor vector

The viral vector was produced as previously described (Pracucci et al., 2023). Briefly, 293AAV cells (Cell Biolabs) were cultured in D-MEM supplemented with 10% FBS, l-glutamine and Pen-Strep. Plates (40 × 150 mm) were transfected using PEI-mediated transfection (Polysciences). For each plate, 1.5 × 10^7^ cells were plated and, on the following day, triple transfected with 30 µg of pAdDeltaF6 helper plasmid (Addgene plasmid #112867), 15 µg of pAAV2-8 Rep-Cap plasmid (Addgene plasmid #112864), and 15 µg of pAAV-EF1a-DIO-ClopHensor. Approximately 16 h after transfection, medium was replaced with D-MEM supplemented with 5% FBS, l-glutamine and Pen-Strep. Cells were collected 72 h after transfection.

The virus was purified as described previously (Potter et al., 2014). The cell pellet was resuspended in 3.7 ml of 39 mM sodium citrate. Cells were then sonicated, and 1 M magnesium chloride was added to a final concentration of 1.6 mM. Benzonase (Sigma-Aldrich) was added at 200 U/ml of cell pellet and incubated 1 h at 37°C. To the crude extract, 5.3 ml of 58 mM citric acid was added to clarify the extract. The extract was then centrifuged at 4,500 × *g* at RT for 10 min. The supernatant was recovered and filtered through a 0.22 µm PES filter (Millipore) and directly applied to a HiPrep SP HP 16/10 column (Cytiva) using an AKTA start (Cytiva) connected to a fraction collector Frac30 (Cytiva). The virus was diafiltrated (1:160,000) and concentrated (to final volume of ∼1 ml) using Protein Concentrators PES, 100 K MWCO (Thermo Fisher Scientific, Pierce).

The final buffer for the virus was dPBS + 5% glycerol + 0.001% Pluronic F-68 (Invitrogen). The AAV titer (1.71 × 10^13^ GC/ml) was determined by qPCR.

### Viral injection

LSSm-ClopHensor was introduced into the neocortical tissue by injecting AAV-viral vectors either in newborn pups (SST-Cre mice) or into adult PV-cre mice. The former was our preferred methodology, because it delivers hemisphere-wide expression but yielded very poor labeling in the PV-cre mice. Importantly though, the mode of introducing LSSm-ClopHensor does not appear to affect the read-out of the biosensor (Pracucci et al., 2023).

For SST-Cre mice, injections of AAV-viral vectors were made into neonatal mice pups [Postnatal Day (P)0–1], followed a previously described protocol (Alfonsa et al., 2015). Briefly, pups were anesthetized with isoflurane (3–4% in O_2_ delivered at 0.8 L/min), and the scalp and skull were perforated using a 23 G needle, ∼1.5 mm in front of the lambda, and roughly 1 mm lateral to the midline, on the left side. A Hamilton Nanofil syringe with a 36 G needle (World Precision Instruments) was then guided through the hole, to a depth of 1.7 mm, aiming for the lateral ventricles, and 250 nl of viral vector was delivered at that depth. Further injections of the same volume were also delivered, at 2 min intervals, at 1.4, 1.1, and 0.9 mm depth. The needle was left in situ for at least 3 min after the final injection. Mice were allowed to recover fully from the anesthetic while being kept warm, before being returned to the home cage. When these mice reached adulthood, a cranial window was created to allow direct visualization of the dorsal neocortex, as previously described (Pracucci et al., 2023). In short, mice were given preoperative analgesia (5 mg/kg Metacam and 0.1 mg/kg buprenorphine) and were then placed into a stereotaxic apparatus under isoflurane anesthesia, 1.5–2% delivered in 95% O_2_ at 0.8–1 L/min. Following incision of the scalp, a 4 mm circular craniotomy was drilled in the left hemisphere. The craniotomy was then closed with a circular glass coverslip and sealed with dental acrylic, and the scalp was sutured, covering the injection areas. Buprenorphine (0.1 mg/kg) was given postoperatively, 6 h after the first dose. The animals were allowed to recover for at least 2 weeks before being used for the imaging experiment.

For PV-Cre mice, viral injections were made during the surgery to create the cranial window. Mice were held in a stereotactic head holder, and injections were made through the craniotomy, at up to three different locations within the primary somatosensory cortex, verified using stereotactic coordinates (Franklin and Paxinos, 2008), in one cortical hemisphere (typically left). At each stereotaxic location, the virus was delivered at multiple depths, using a Hamilton nanofil syringe needle (36 G, World Precision Instruments; 500 nl of virus at 0.7 and 0.4 mm deep to the pial surface), with the needle left in situ for 2–5 min following each viral evacuation. The craniotomy was then closed with a circular glass coverslip as described above, and again, the animals allowed to recover for at least 3 weeks to allow expression levels to build up. A custom-made titanium headplate was cemented to the skull of the mice to allow positioning under the two-photon microscope using a custom-made head holder. Mice were used for the imaging steps only if no scar tissue was present in the window and if the implant was stable and did not require additional repairs nor replacement. Mice showing evidence of cortical damage or bleeding caused by abrasions to the dura during preparation were excluded.

To allow all imaging experiments to be performed during the working day, we moved those allocated to the ZT17 group onto a reversed light cycle for at least 1 week prior to imaging (and sometimes up to 1 month). Prior to the imaging sessions, each mouse was moved to a new cage and its activity monitored for at least 24 h to verify a normal activity pattern (which typically stabilized ∼4 d after transferring to the new light cycle). An automated monitoring system was used, consisting of an IR camera (ELP KL36IR 1080P Webcam) controlled by a custom MATLAB script that tracked the mouse's locomotion. As expected, all mice, including those moved on a reversed light cycle for >4 d, showed increased activity during the dark phase of their light cycle [data not shown, but examples can be found in Pracucci et al. (2023)]. Daily animal checks were made soon after the lights turned on to minimize disruption to their circadian behavioral cycles.

### In vivo two-photon imaging

All imaging experiments were performed as acute terminal experiments on adult mice aged 2–10 months, which had been injected previously with LSSm-ClopHensor. Mice were anesthetized with isoflurane (inhalation, 4–5% in 0_2_ for induction, then lowered to 1.5% at the beginning of the procedure and gradually discontinued before starting the imaging session) and urethane (1.5 g/kg, i.p., with additional supplement of 0.15 g/kg after 1 h if needed). Once mice were fully anesthetized, they were injected with 0.015 mg/kg glycopyrrolate subcutaneously, to reduce respiratory mucus secretions, and then positioned in the stereotaxic frame. Isoflurane was used only for induction; maintenance of anesthesia was achieved using only urethane and assayed by checking the pedal withdrawal reflex; imaging data collection was commenced at least 20 min after suspension of isoflurane administration. Mice were positioned in a mask supplying oxygen for the entire duration of the experiment, and their body temperature was monitored and maintained stable (above 35°C) using a heated pad connected to a rectal probe. Between 3 and 5 fields of view (FoVs) were acquired for each mouse.

### Two-photon acquisition parameters

All imaging sessions were performed on a Thorlabs Bergamo II 2-Photon microscope, equipped with a BB MaiTai laser (Spectra-Physics) and a Nikon 16× lens [NA 0.8 (water immersion)]. Following excitation at the experimental wavelengths (830, 860, 910, 960 nm), emitted fluorescence was collected in the green and red channels via two photomultiplier tubes equipped with Chroma filters: 527/70 nm and 607/70 nm, respectively, dichroic 562 nm. Acquisitions were performed at a resolution of 512 × 512 pixels and at zoom 2× (field of 414.65 × 414.65 µm) or 4× (206.88 × 206.88 µm). Different wavelength acquisitions were performed at equivalent power, as measured immediately below the objective using a Thorlabs power meter (Thorlabs, PM100D and S470C; power checked weekly).

For each FoV, the lens position was set to 0 at the surface of the pia, and a *Z*-stack was acquired starting ∼60–80 µm deep (where the first cell bodies could be identified) up to 250–300 µm deep, in 7–10 µm steps. Each focal plane acquired was the result of a 10× cumulative averaging.

### Two-photon image processing

All the two-photon imaging data were collected as TIFF Files (.tif) through the ThorImage software (Thorlabs). Analysis of the images was performed using a custom MATLAB script (MathWorks) and based on the workflow previously published (Sulis Sato et al., 2017; Pracucci et al., 2023). Briefly, the following corrections/computations were applied to each dataset:

1. Dark frame/residual light subtraction: dark images were taken for each imaging session by acquiring a frame when the laser was off, to monitor the electrical noise of the photomultiplier tubes and the residual light in the room. The frame was acquired using the same settings used for the experiments, and the mean dark values of each channel value were subtracted from the imaging data.
2. Flat field correction: the acquisitions of a rhodamine 6G aqueous solution have been used to correct for artifacts introduced by optical components, such as higher fluorescent intensity values at the center of the image compared with the edges and corners.
3. Image registration: for each set of images, the script performs a 2D cross-correlation between a reference frame (acquisition of the red fluorescence at 910 nm) and the frames acquired at the other wavelengths. This allows to correct for any drift on the *x*–*y* direction during the acquisition.
4. Automatic cell identification: the code automatically detects cells as the brighter areas in the FoV and then filters each of them for specific parameters including the size, morphology, variance, and signal-to-noise ratio (SNR).
5. pH and [Cl^−^]_in_ computation: for each identified cell, the values of green and red fluorescence are computed. As reported before (Sulis Sato et al., 2017), these values are corrected for an additional series of parameters, such as depth, scatter, and intracellular environment. The code then computes the ratio between the emission from the green channel divided by the red channel (G/R) and calculates intracellular pH and [Cl^−^]_in_ estimates for each cell, based on the calibration experiments on ionophore-permeabilized HEK cells (Sulis Sato et al., 2017).

### Immunolabeling of LSSm-ClopHensor in brain slices

At the end of the terminal imaging session, mice were intracardially perfused with 4% paraformaldehyde (PFA) in PBS (Santa Cruz Biotechnology). The brain was dissected and stored in 4% PFA. Before sectioning, the brain was immersed in a solution of 30% sucrose in PBS and allowed to equilibrate overnight. Coronal sections (40 µm) were cut using a freezing microtome (8,000 Retracting Sledge Microtome, Bright Instruments). Immunohistochemistry to label LSSm-ClopHensor (modified E^2^ GFP domain), PV, and SST was performed as previously described (McLeod et al., 2017). Briefly, following antigen retrieval (10 mM Na-citrate buffer) pH 6, slices were incubated (3 h at 23°C) in blocking buffer (10% donkey serum, 0.05% Triton X-100 in PBS). Slices were then incubated overnight, at 4°C, in blocking buffer containing the primary antibodies. After three washes in PBS, slices were incubated 2–3 h, at 23°C, in blocking buffer containing secondary antibodies. After three washes in PBS the slices were mounted on gelatin-coated glass slides using Fluoromount-G mounting media (Sigma-Aldrich). Antibody dilutions used are reported in Table 1.

**Table 1.** Details of the antibodies used for the immunolabeling experiments

Antibodies	Concentration
SST (D-20): sc-7819 (Santa Cruz Biotechnology), Goat	1:200
PV: PV27 (Swant), Rabbit	1:2,000
GFP: ab13970 (Abcam), Chicken	1:1,000
647-anti-goat: A21447(Invitrogen), Donkey	1:500
568-anti-rabbit: A10042 (Invitrogen), Donkey	1:500
488-anti-chicken: 703-545-155 (Jackson Immuno), Donkey	1:500

Confocal images were acquired on a Zeiss LSSM880 upright microscope using a water immersion 40× lens: W Plan-Apochromat 40×/1.0 DIC VIS-IR M27 (Newcastle University, Bioimaging Unit). *Z*-stacks (steps of 1–1.5 µm) were acquired in the cortical region of the slices with a 512 × 512 pixel resolution. All images were acquired in the superficial layers of the cortex, at a depth compatible with the two-photon images acquisition (not deeper than 250–300 µm from the pial surface). At least four slices per condition were examined. All image processing and analyses were performed using Fiji (Schindelin et al., 2012). For *Z*-stack maximum projections, brightness/contrast adjustments were fixed within the individual channels.

### Brain slice recordings

Brain slices were prepared from young adult mice expressing channelrhodopsin-2 (ChR2) under the Emx1 promoter, which, in adult mice, yields high expression of ChR2 selectively within neocortical pyramidal cells (Gorski et al., 2002; Parrish et al., 2023). Prior to experimentation, mice were kept in quiet rooms, on 12 h light/dark cycles, for at least 3 d, with cages being checked just once a day, to minimize disturbance to the mice. On the day of experimentation, mice were taken from their home cage and immediately anesthetized by intraperitoneal injection of ketamine (100 mg/kg)/medetomidine (1 mg/kg). Injections performed within 60 s of initial disturbance of animals, while still in the animal housing area and prior to being taken to the laboratory. This was done either at ZT5 or ZT17. Once fully anesthetized, mice were perfused through the heart with a modified, osmotically matched sucrose solution (in mM: 228 sucrose; 26 NaHCO_3_; 3 KCl; 1.25 NaH_2_PO_4_; 4 MgCl_2_; 10 glucose). The brain was removed, and 350 µm coronal slices were cut in this same sucrose solution. Slices were then transferred to an incubation chamber containing conventional artificial cerebrospinal fluid solution (aCSF; in mM: 126 NaCl; 26 NaHCO_3_; 3.5 KCl; 2 CaCl_2_; 1 MgCl_2_; 1.26 NaH_2_PO_4_; 10 glucose), where they were kept moist at the interface between the solution and air, at room temperature. All solutions were bubbled with carbogen (95% O_2_, 5% CO_2_) throughout.

For the recordings, slices were transferred to a recording interface chamber, being perfused with aCSF at 2–3 ml/min and kept at 32–35°C. A Neuronexus multielectrode probe, with a single recording site on each of 8–16 prongs (100 µm separation of prongs), was inserted into the brain slice at its dorsal pole, oriented orthogonal to the pial surface (Fig. 5*A*). Optogenetic stimulation was delivered using a Thorlabs LED controller (M470F3) to direct a temporally modulated spot (∼1 mm diameter) of blue light (470 nm) onto Layer 1, ∼1–2 mm lateral to the electrodes, using an optic fiber that was pointing away from the electrodes, to avoid driving any nonbiological photoelectric currents. The timing of illumination was controlled using the Spike2 software generating TTL commands through a 1401 AD board (Cambridge Electronic Design). The first assay involved delivering a train of 10 optogenetic stimuli (10 ms duration) at 10 Hz, while the second assay used optogenetic ramps, with the light monotonically increasing from zero to maximal, over 1 s. Field recordings were sampled at 20 kHz, filtered at 0.1–7,500 Hz, using an Intan 512 channel recording controller. Data were analyzed offline using the software written in MATLAB (MathWorks). Current source density analysis used code written by Timothy Olsen (https://www.mathworks.com/matlabcentral/fileexchange/69399-current-source-density-csd, Retrieved September, 2023, through the MATLAB Central File Exchange), based on prior published descriptions of this analysis technique (Nicholson and Freeman, 1975; Pettersen et al., 2006).

### Statistical analysis

All the statistical analyses described were performed using MATLAB 2020b-2022a inbuilt functions. All datasets were checked for normality using a one-sample Kolmogorov–Smirnov test. If samples were normally distributed, statistical comparisons were made using Student's *t* tests (MATLAB, ttest2.m); otherwise, we used Mann–Whitney rank sum tests (MATLAB, ranksum.m). Differences were considered significant if *p* < 0.05.

## Results

### Cell-class–specific in vivo expression of LSSm-ClopHensor

**Figure 1.**
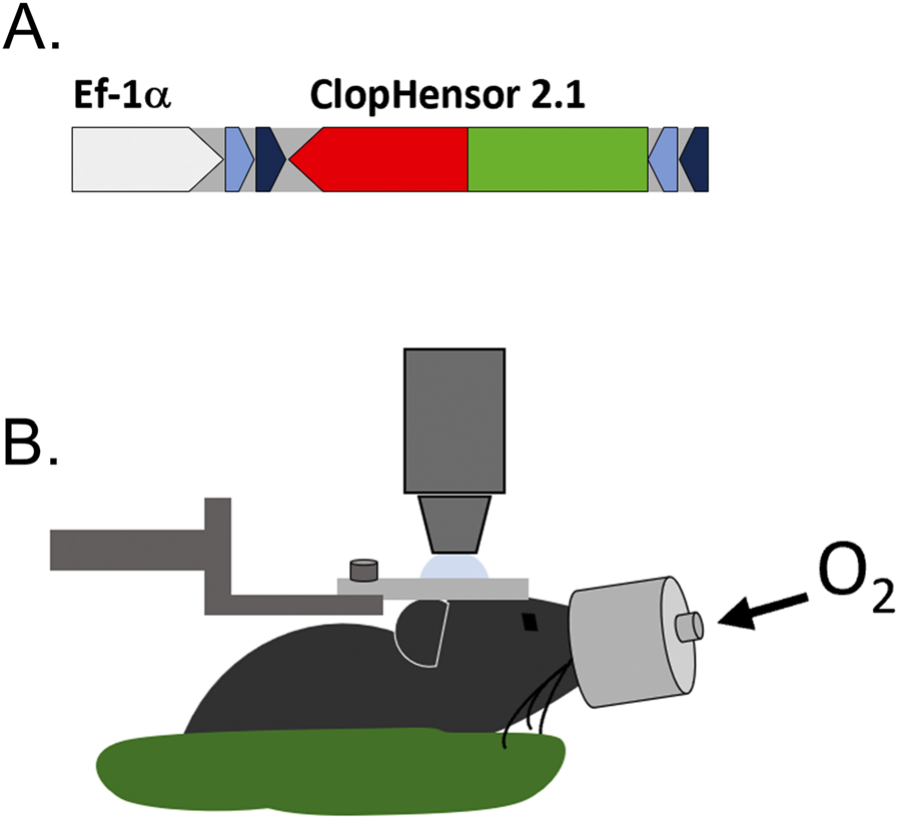
In vivo expression of LSSm-ClopHensor 2.1 in interneurons. ***A***, Schematic of the construct packaged into AAV vector for cell-specific targeting. An inverted LSSm-ClopHensor sequence (red and green) is inserted between two pairs of Lox sites (blue) and under the control of EF1α promoter (white). ***B***, Schematic of the experimental setup for the imaging session: the anesthetized mouse (urethane only) is held in place by a custom-made system consisting in a cemented headplate secured to a post. For the duration of the experiment, the mouse is positioned on a heated pad (green) with a feedback probe and is provided oxygen through a face mask.

**Figure 2.**
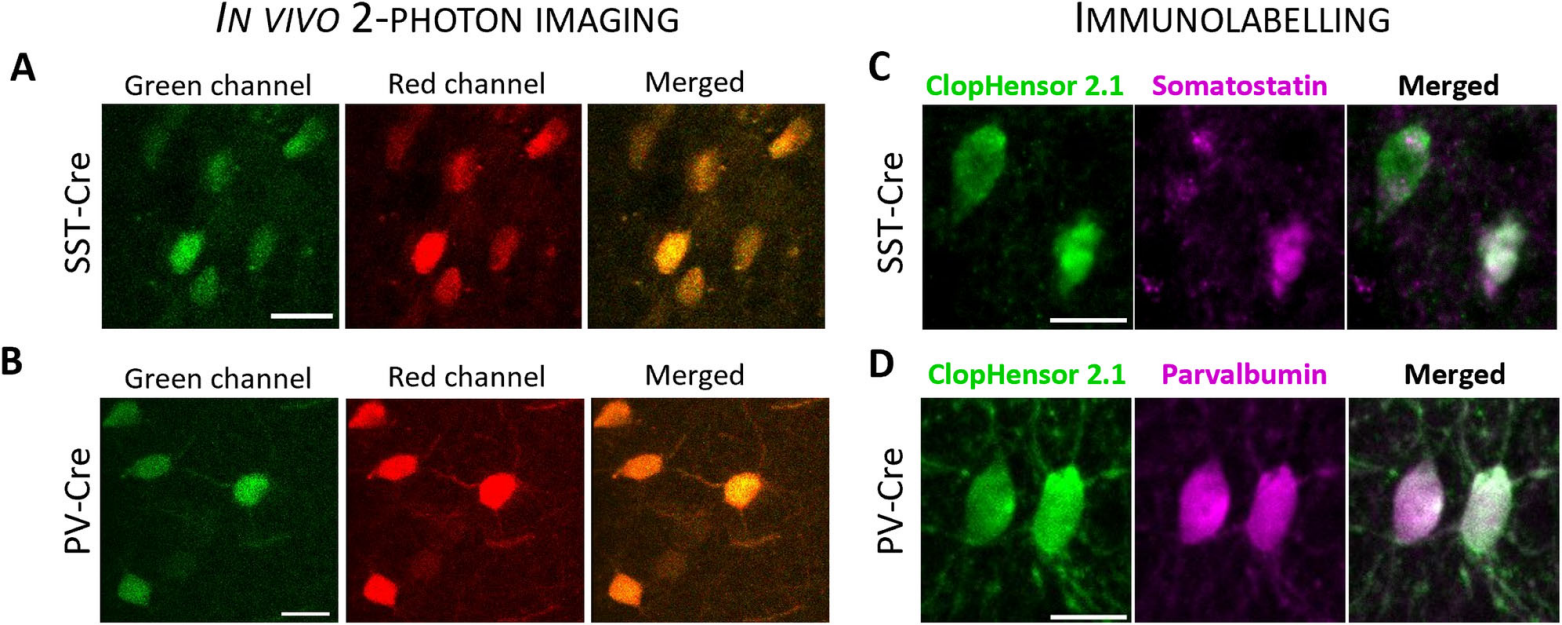
Cell-class–specific LSSm-ClopHensor expression in SST-cre and PV-cre mice. ***A***, Representative in vivo two-photon images of SST and (***B***) PV interneurons, expressing LSSm-ClopHensor following viral injection. Fluorescence in the green channel (left panel), red channel (middle panel), and merged signals (right panel) are shown following excitation at 910 nm. Each set of images is the maximal projection of three focal planes acquired during a *z*-stack (10 µm *z*-steps). Scale bars, 20 µm. ***C***, Immunohistochemistry for LSSm-ClopHensor (green) and SST (magenta) in an SST-Cre mouse injected with AAV8Ef1aDIOClopHensor. The labeling confirms that the sensor is expressed in SST^+^cells (merged). Scale bar, 10 µm. ***D***, Immunohistochemistry for LSSm-ClopHensor (green) and PV (magenta) performed on a PV-Cre mouse injected with AAV8Ef1aDIOClopHensor. The labeling confirms that the sensor is expressed in PV cells (merged). Scale bar, 15 µm.

Following on from our recent observation of unexpectedly large physiological modulation of [Cl^−^]_in_ in mouse neocortical pyramidal cells through the day (Pracucci et al., 2023), we used the same imaging techniques to examine whether other classes of cortical neuron show similar [Cl^−^]_in_ dynamics. Population analyses of intracellular [Cl^−^]_in_ and pH_in_ were performed in adult mice, in the two largest subpopulations of neocortical interneurons, identified by conditional expression of the genetically encoded biosensor, LSSm-ClopHensor, under either the PV or SST promoters. To achieve targeted expression in specific interneuronal populations in vivo, we injected a viral vector, carrying the floxed sequence of LSSm-ClopHensor, into the somatosensory cortex (S1) of mice expressing Cre-recombinase (Gaveriaux-Ruff and Kieffer, 2007) under those promoters (Fig. 1*A*). This yielded interneuronal cell-class–specific labeling of LSSm-ClopHensor expression (Fig. 2*A*,*B*), sustained for up to 12 months following injection of the viral vector. To confirm the patterns of expression, we performed immunohistochemical labeling using antibodies against GFP, which binds also to the modified E^2^GFP component of LSSm-ClopHensor, which colocalized precisely to expression also of PV (Fig. 2*C*) or SST (Fig. 2*D*), in the two mouse lines, respectively.

### Differences in intracellular pH in cortical interneurons

**Figure 3.**
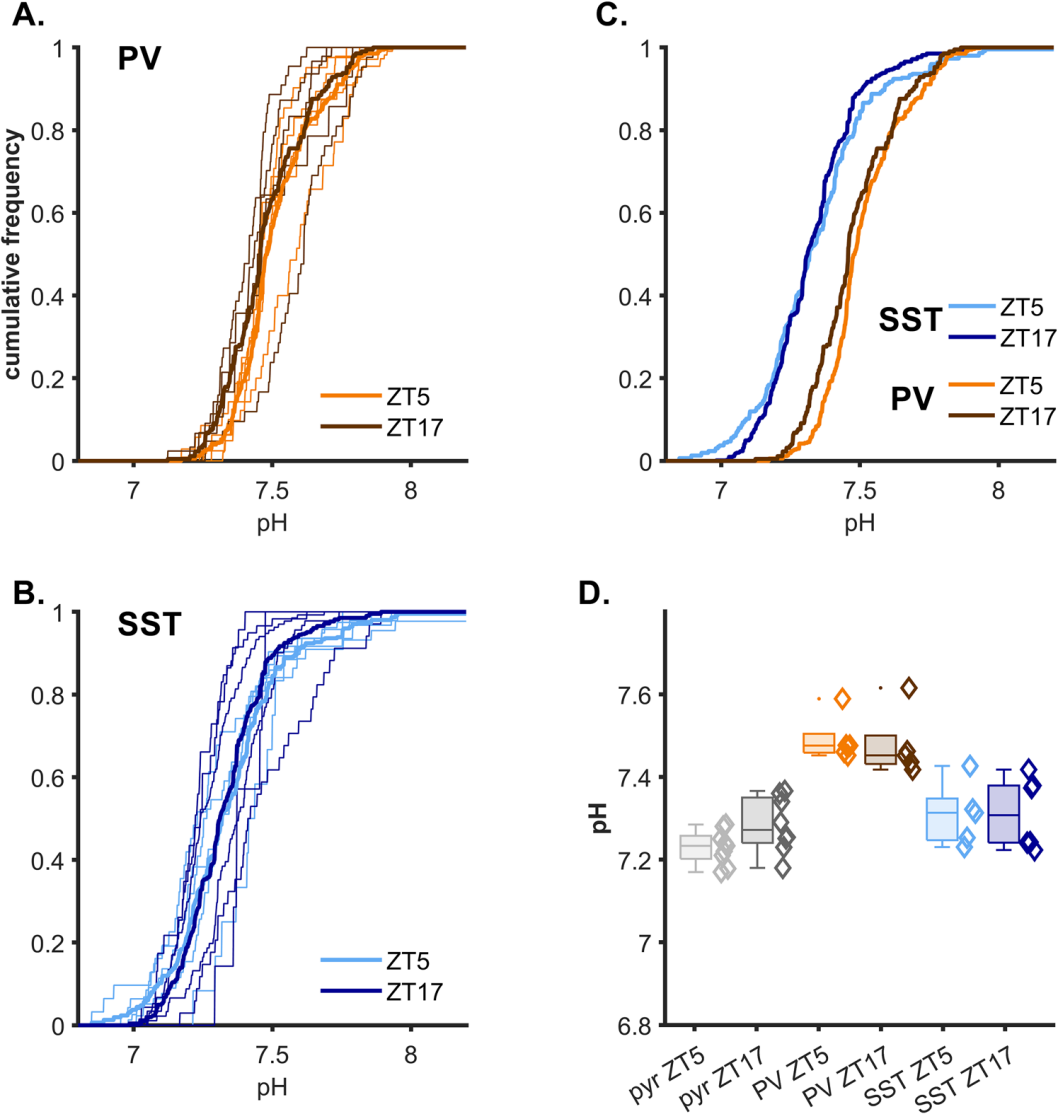
PV and SST interneurons exhibit differences in their intracellular pH. ***A***, Cumulative frequency plot of the distribution of pH_in_ values for all neurons, in each PV-Cre mouse (thin lines) during the day (ZT5, orange, 5 animals) and at night (ZT17, brown, 5 animals). Each line represents a single animal. The thick lines show the average values for the two time points. ***B***, Cumulative frequency plot of the pH_in_ values obtained from SST-Cre mice. Thin lines represent the distribution of each mouse acquired during the day (ZT5, light blue, 5 animals) or during the night (ZT17, dark blue, 6 animals). The thick lines show the averaged distribution pooled from all the animals examined at ZT5 and ZT17, respectively. ***C***, Direct comparison of the distribution of the averaged pH_in_ cumulative distributions (reproduced thick lines in panels ***A*** and ***B***). ***D***, Median values (diamonds), together with the median/upper/lower quartile ranges for animals across groups, for the two time points for the Layer 2/3 pyramidal cells from our previous publication (Pracucci et al., 2023) and the two interneuron populations presented here.

Having validated the expression systems, we performed in vivo LSSm-ClopHensor two-photon imaging, under urethane anesthesia, at two time points, ZT5 and ZT17, chosen because these corresponded to the times when pyramidal [Cl^−^]_in_ showed the peak and minimum levels in our previous study (Pracucci et al., 2023). Analysis of fluorescence of the double-ratiometric biosensor, LSSm-ClopHensor, involves first deriving the pH_in_ measures (Arosio et al., 2010; Sulis Sato et al., 2017). Since two factors were being considered simultaneously (cell class and time of day), we analyzed the data using a two-way ANOVA, with Bonferroni’s corrections for multiple testing (MATLAB, anovan.m, multcompare.m; Table 2, Fig. 3). The simple main effects analysis revealed that cell class had a highly significant effect on pH_in_ (*F*_(2,37)_ = 39.2; p≪0.001), but there was no significant effect of time of day (*F*_(1,37)_ = 0.44; *p* = 0.514) and no significant interaction between cell class and time of day (*F*_(2,37)_ = 0.96, *p* = 0.393). Pairwise comparisons found that pH_in_ in PV interneurons was significantly more alkaline than in the other two cell classes, but there was no significant difference in pH_in_ between SST and pyramidal cells (Table 2).

**Table 2.** pH measurements in and comparisons between neocortical neuronal subclasses

	ZT5	ZT17
Pyramidal cell (Pracucci et al., 2023)	7.23 ± 0.04 (9, 663)	7.28 ± 0.07 (8, 1,054)
PV interneuron	7.49 ± 0.06 (5, 249)	7.48 ± 0.08 (5, 177)
SST interneuron	7.31 ± 0.08 (5, 212)	7.31 ± 0.09 (6, 348)

Each line gives mean ± standard deviation and, in parentheses, number of animals and cells.

### Divergent temporal modulation of [Cl^−^]_in_ in neocortical neuronal classes

**Figure 4.**
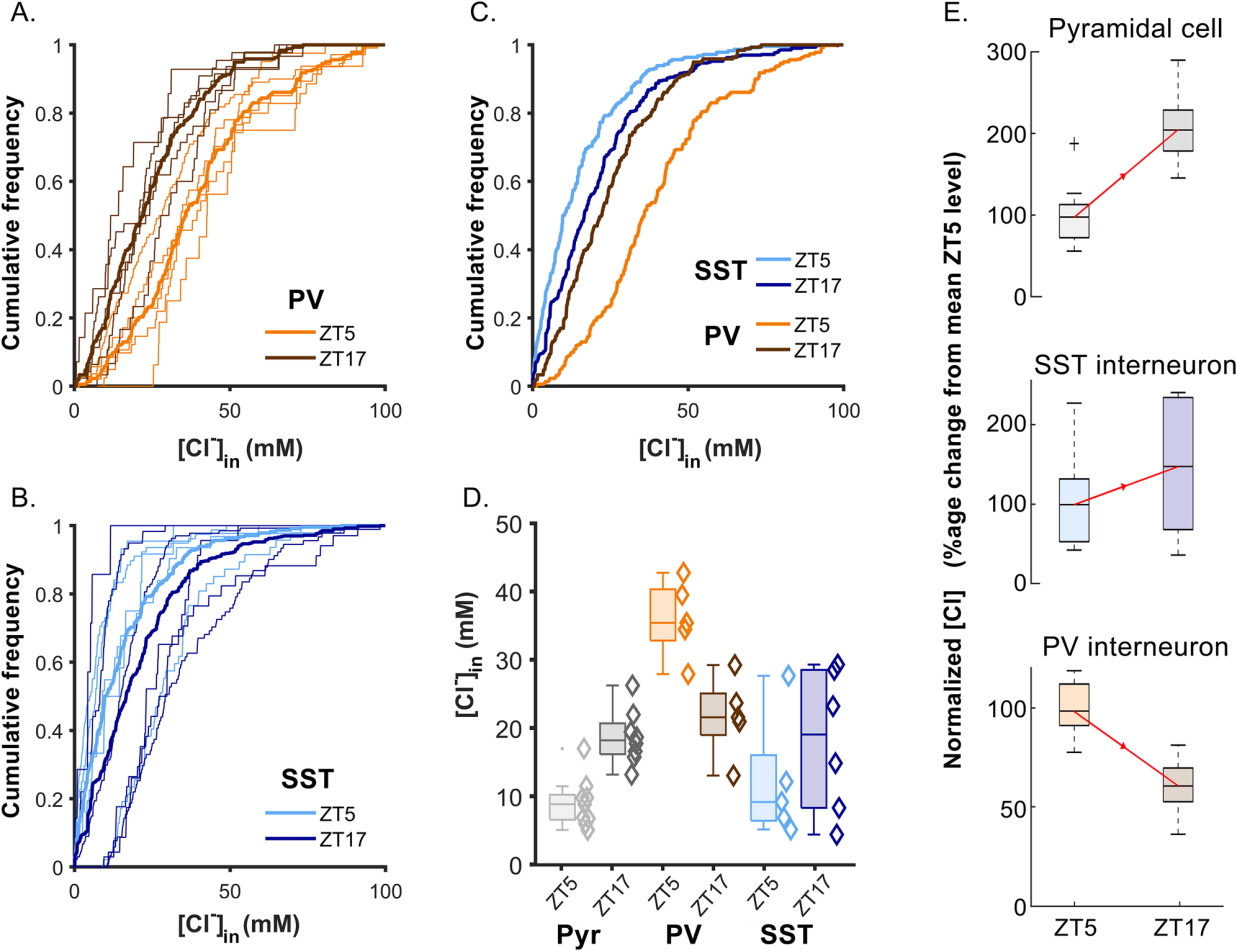
Comparison of the intracellular chloride values of PV and SST interneurons. ***A***, Cumulative frequency plot of the distribution of [Cl^−^]_in_ values for all neurons, in each PV-Cre mouse (thin lines) during the day (ZT5, orange, 5 animals) and at night (ZT17, brown, 5 animals). Each line represents a single animal. The thick lines show the average values for the two time points. ***B***, Cumulative frequency plot of the [Cl^−^]_in_ values obtained from SST-Cre mice. Thin lines represent the distribution for all neurons, in each mouse acquired during the day (ZT5, light blue, 5 animals) or during the night (ZT17, dark blue, 6 animals). The thick lines show the averaged distribution pooled from all the animals examined at ZT5 and ZT17, respectively. ***C***, Direct comparison of the distribution of the averaged [Cl^−^]_in_ cumulative distributions (reproduced thick lines in panels ***A*** and ***B***). ***D***, Median values (diamonds), together with the median/upper/lower quartile ranges for animals across groups, for the two time points for the Layer 2/3 pyramidal cells from our previous publication (Pracucci et al., 2023) and the two interneuron populations presented here. ***E***, The same data shown in ***D*** but normalized to the mean values at ZT5, shown to highlight the different trajectories of [Cl^−^]_in_ modulation in PV interneurons and the other two classes.

We next determined [Cl^−^]_in_ at the two time points, for both interneuron populations, using the same imaging dataset (Table 3, Fig. 4), and analyzed again using a two-way ANOVA. We found highly significant differences in [Cl^−^]_in_ between cell classes (*F*_(2,37)_ = 18.3; p≪0.001) and also a highly significant interaction between cell class and time of day (*F*_(2,37)_ = 11.2; *p* = 0.002), indicating that the pattern of modulation differed between cell classes. This is most clearly seen in the opposite direction of modulation of [Cl^−^]_in_ seen in PV interneurons and pyramidal cells, from ZT5 to ZT17: pyramidal [Cl^−^]_in_ rose over this period, while in PV interneurons, it dropped. When each class was analyzed individually, both PV interneurons and pyramidal cells showed highly significant changes between ZT5 and ZT17 [Student's *t* tests, PV, ZT5 vs ZT17, *p* = 0.0042; pyramidal, ZT5 vs ZT17, *p* = 0.00011 Pracucci et al. (2023)]. In SST interneurons, [Cl^−^]_in_ measures showed much greater variance than was found in the other two cell classes (Table 3, Fig. 4), and so the two time points did not show a significant difference (*p* = 0.351, Student's *t* tests), although interestingly, the trend from day to night appears more consistent with the pattern of pyramidal cell modulation than with PV interneurons (Fig. 4*E*) . Reflecting this apparent divergent modulation within the two interneuronal classes, we found that PV and SST interneuron [Cl^−^]_in_ measures were significantly different at ZT5 (*p* = 0.001, Student's *t* tests), but not at ZT17 (*p* = 0.514, Student's *t* tests). Notably, there was no correlation between intracellular pH and [Cl^−^] in any cell class (Table 4).

**Table 3.** [Cl^−^]_in_ measurements in cortical neurons

	ZT5	ZT17
Pyramidal cell (Pracucci et al., 2023)	9.1 ± 3.6 (9, 663)	18.7 ± 4.1 (8, 1,054)
PV interneuron	36.0 ± 5.6 (5, 249)	21.7 ± 5.8 (5, 177)
SST interneuron	12.2 ± 9.0 (5, 212)	18.1 ± 10.5 (6, 348)

Each line gives mean ± standard deviation and, in parentheses, the numbers of animals and cells.

**Table:** 

Pairwise comparisons	Difference between means (95% confidence interval)	*p* value
Pyramidal v PV	0.227 (0.160–0.294)	2.8 × 10^−9^
Pyramidal v SST	0.054 (−0.011–0.120)	0.133
PV v SST	−0.173 (−0.099 – −0.247)	4.1 × 10^−6^

**Table 4.** Intracellular pH and [Cl^−^] are not correlated

	ZT5		ZT17	
	*R* ^2^	*P*	*R* ^2^	*p*
Pyramidal cells	0.024	0.693	0.004	0.876
PV interneurons	0.06	0.692	0.054	0.706
SST interneurons	0.065	0.678	0.149	0.45

### Time-dependent differences in cortical inhibition persist in vitro

**Figure 5.**
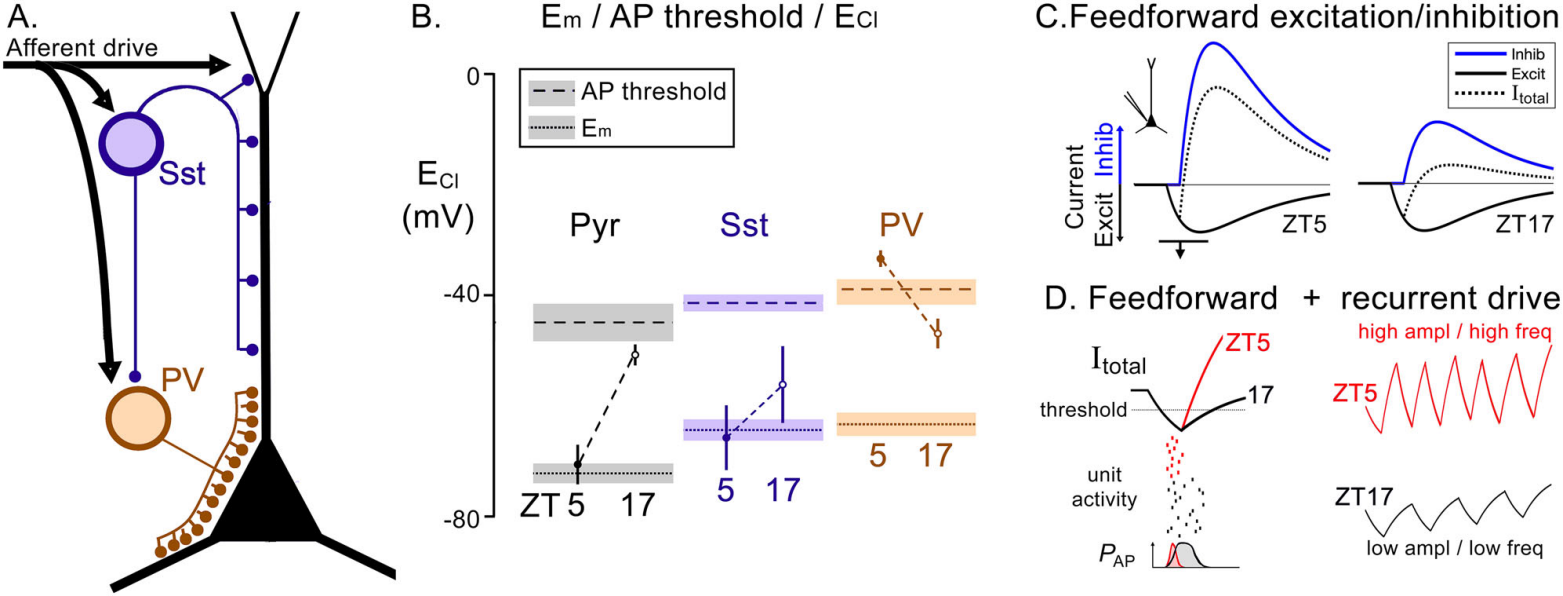
Schematic illustration of the network effects of altered neuronal [Cl^−^]_in_. ***A***, Connectivity diagram for the three classes of cortical neuron examined [based on Pouille and Scanziani (2001); Pfeffer et al. (2013)]. ***B***, Comparison of estimates of the chloride reversal potential, ECl [calculated from our measurements of [Cl]_in_ (mean ± SEM) and assuming [Cl]_out_ = 130 mM] with in vitro measures of membrane potential, Em, and action potential threshold made from the same cell classes (Codadu et al., 2019). ***C***, Schematic showing the sequential feedforward excitatory and inhibitory currents seen in pyramidal cells [based on Pouille and Scanziani (2004)]. The inhibitory component is predicted to be greatly enhanced at ZT5 for two reasons: first that PV interneurons are relatively disinhibited and GABA may even be excitatory because *E*_Cl_ appears to be above action potential threshold (panel ***B***) and, second, that pyramidal [Cl]_in_ is very low, and so the postsynaptic GABA effect in pyramidal cells is relatively hyperpolarizing. ***D***, The top-left panel shows an enlarged view of the combined excitatory–inhibitory currents (*I*_Total_), which create a “window of opportunity” for pyramidal cell firing (Pouille and Scanziani, 2004). In the ZT5 case, this will be shorter and more sharply curtailed which should reduce jitter in the postsynaptic firing at ZT5; this is illustrated schematically in the bottom panel, following the findings of Alfonsa et al. (2015). Tighter coordination of pyramidal firing will then boost the subsequent excitatory drive; if this is being fed back into the network as a recurrent pathway, the combined effect of enhanced excitation with a rapid curtailing of firing each iteration should lead to oscillations of increased power and frequency (right panel).

The large modulation of [Cl^−^]_in_ will dramatically alter *E*_GABA_ in the different cortical classes throughout the day, with secondary consequences for all manner of cortical processing and signaling [Fig. 5; see also Pracucci et al. (2023)]. At ZT5, we predict that PV interneurons will be relatively disinhibited, while their output at that time will strongly inhibit pyramidal cells. In contrast, at ZT17, PV interneurons will experience more effective GABAergic restraint of their firing but may also receive more excitatory drive, because the pyramidal cells will be disinhibited. As noted earlier, SST [Cl^−^]_in_ modulation shows trends more in common with the pyramidal pattern (Fig. 4*E*), although the day/night difference was not significant. These fluctuations in the relative excitability of different neuronal populations should alter the dynamic interplay between inhibitory and excitatory populations. To test this prediction, we used two different experimental assays of inhibition within the supragranular neocortex, in brain slices prepared from mice expressing ChR2 in pyramidal neurons under the Emx1 promoter and which were killed either at ZT5 (17 brain slices) or ZT17 (16 brain slices). Brain slices are relatively quiescent, and so the response to stimulation may be examined independent of ongoing rhythmic activity arising either from other external influences or from internal pattern generators that is the case in vivo. Importantly, previous work had shown that altered levels of pyramidal [Cl^−^]_in_ at different times of day persist following brain slicing (Alfonsa et al., 2023).

**Figure 6.**
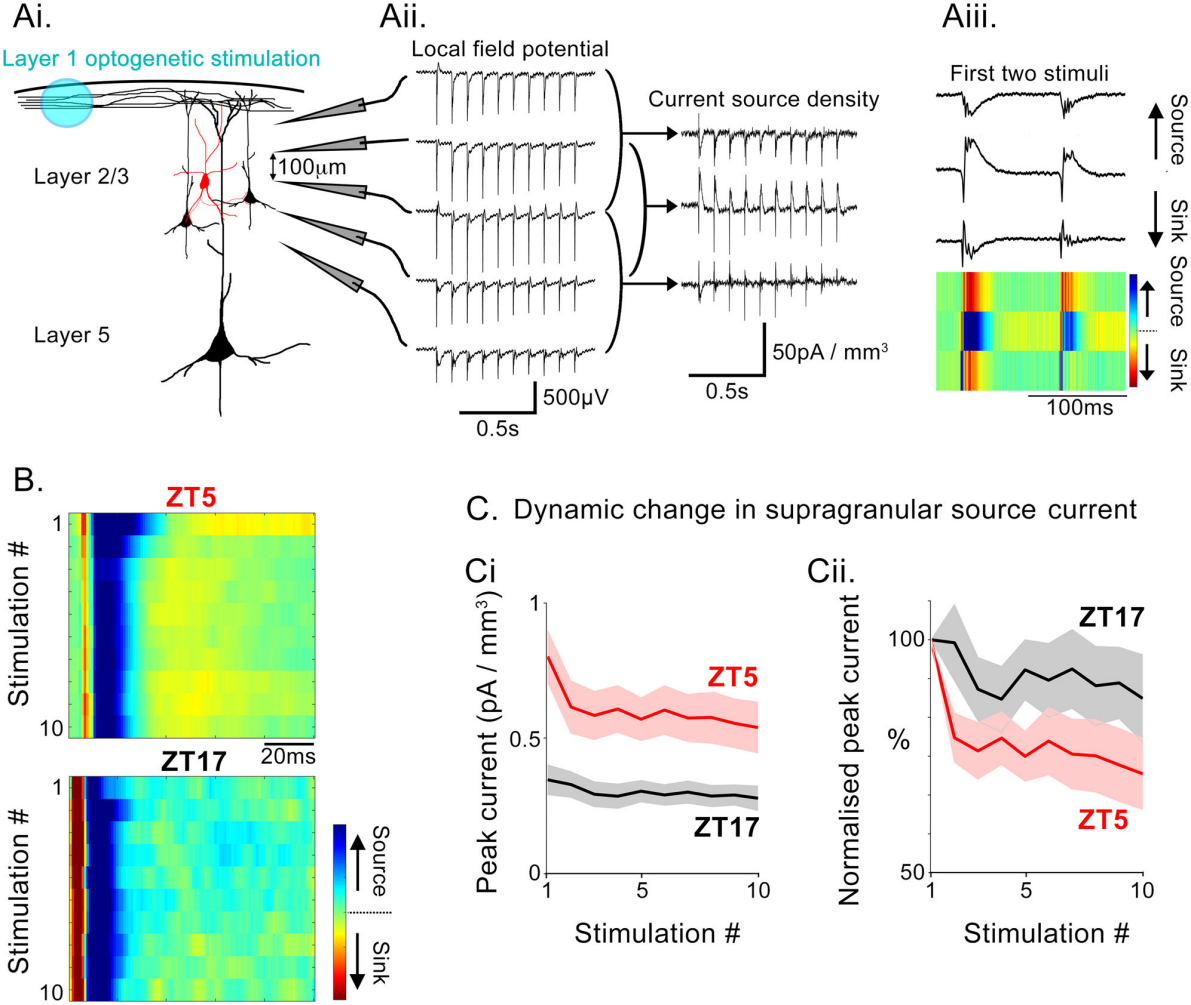
Day/night differences in assay of cortical feedforward inhibition, in vivo. ***Ai***, Schematic showing the experimental arrangement of the optic fiber and the multielectrode arrays in the brain slice. ***Aii***, Example traces of local field potentials during a train of stimuli and the derived current source densities. ***Aiii***, Enlarged view of CSDs for the first two stimuli and a color-coded representation of the CSDs, showing that the sink-source sequence is generally found only in a single electrode in the supragranular layers. ***B***, Color-coded representation of each stimulus in a train of 10 (delivered at 10 Hz). ***C***, The peak of the inhibitory (“source”) current for data collected from ZT5 (red) and ZT17 (black) brain slices, showing highly significant differences at the two times, both for the absolute peak values and (***Ci***) the pattern of adaptation from the first pulse in the train (***Cii***).

The first inhibitory assay examined feedforward inhibition in response to a 10 Hz train of 10 optogenetic stimuli (10 ms light flashes) delivered to Layer 1, recording the extracellular field potential responses across the cortical layers, from the pia to the white matter plane. This pattern of stimulation generated a large amplitude current sink-source sequence within the supragranular layers (Fig. 6*A*) that was identified using current source density analysis (Nicholson and Freeman, 1975; Pettersen et al., 2006). This is indicative of an initial glutamatergic drive (the “sink”) being curtailed by rapid feedforward inhibition (the “source”), similar to that described in the hippocampus (Pouille and Scanziani, 2004; see also explanatory schematics in Fig. 5). Pouille and Scanziani further described how the source of inhibition evolves during the course of sustained stimulation, initially being provided by PV interneurons but switching thereafter to deriving from SST interneurons. We found significant differences between brain slices prepared from mice killed at the two different ZT times (Fig. 6). In brain slices from ZT5 preparations, the initial peak inhibitory current was significantly larger (Fig. 6*C*; *p* = 0.0004, Mann–Whitney rank sum test). The 26.4% drop in the amplitude of the source current between the first and second pulse of the trains in ZT5 brain slices was highly significant (null hypothesis, 0% change; 95% confidence interval, 36.0–14.5%). These changes appear to be consistent with predictions of how the [Cl^−^]_in_ affects activity in the two interneuronal populations. At ZT5, disinhibited PV interneurons create a powerful immediate feedforward inhibition of pyramidal cells. However, the subsequent buildup of SST activity is delayed, because those cells have more negative *E*_GABA_, and their activity is thus restrained. Thus, the large drop between the first and second pulses, at ZT5, reflects the relative dominance of the PV-to-SST inhibitory effects. In contrast, there was no change between the first and second peaks in the ZT17 brain slices (normalized amplitude of the second peak, 99.2%; ZT5 vs ZT17, *p* = 0.026, Student's *t* test), consistent with a relatively weaker initial activation of PV interneurons and an earlier transition to SST activation.

**Figure 7.**
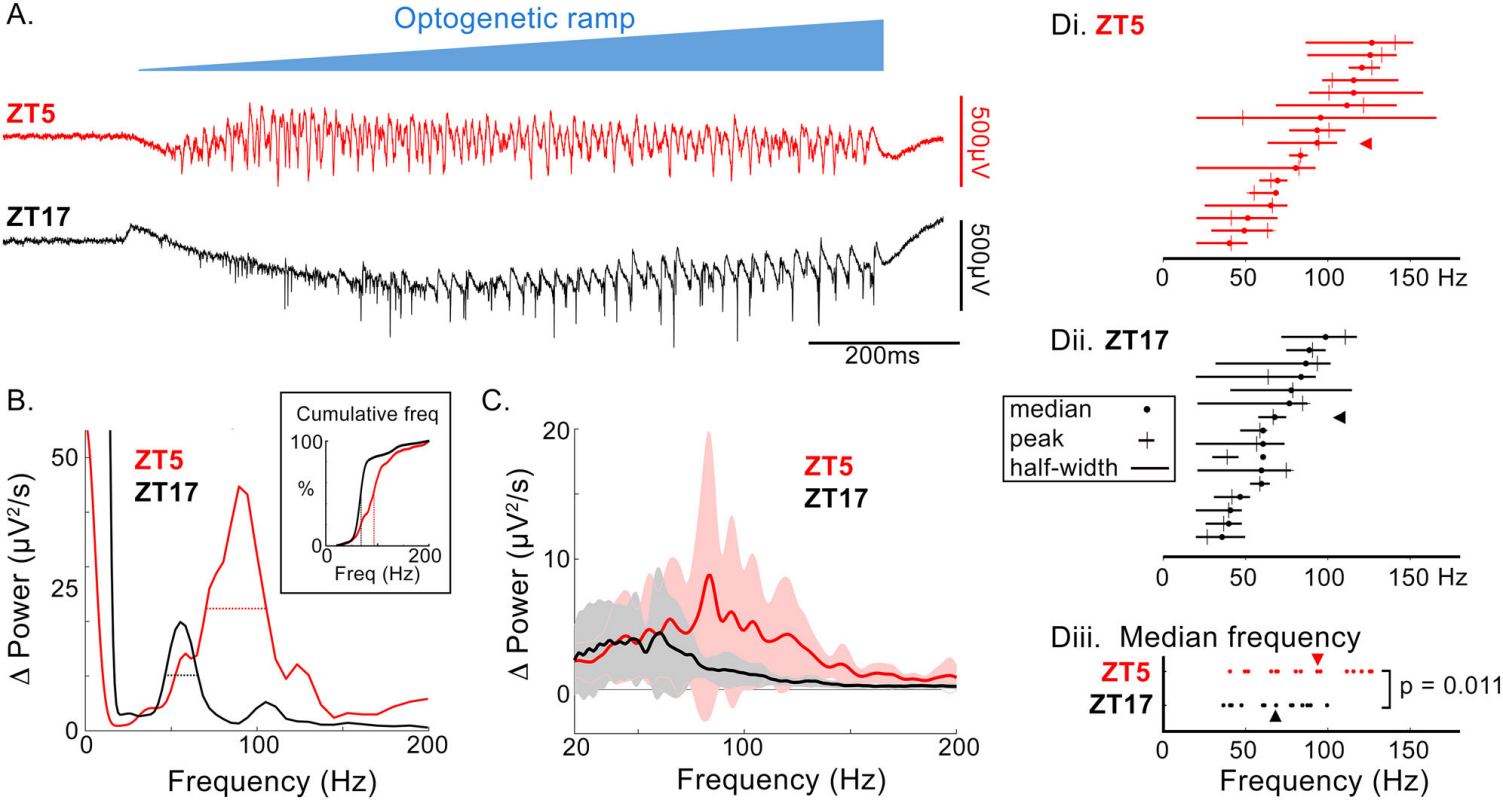
Day/night differences in optogenetically induced brain oscillations. ***A***, Representative traces showing oscillations induced by an optogenetic ramp to the adjacent cortical territory (see Fig. 6*A* for experimental arrangement). ***B***, The increase in power for the traces shown in ***A***, relative to an equivalent duration epoch immediately prior to the ramp onset. The horizontal dotted line shows the half-width calculation; the inset shows the cumulative power between 20 and 200 Hz, indicating how the “median frequency” was calculated. ***C***, The same plot for all 33 brain slices (17 ZT5 slices; 16 ZT17 slices; thick line represents mean, and the shaded area represent the 95% confidence interval). ***D***, The half-width (horizontal lines), median (filled circles), and peak (cross-hairs) frequencies for each brain slice ordered by the median frequency (***Di***, red, ZT5; ***Dii***, black, ZT17). ***Diii***, distribution of medians for ZT5 and ZT17 slices, shown side-by-side. The red and black arrowheads indicate the example shown in panels ***A*** and ***B***.

The second assay used the same physical arrangement of optic fiber and multielectrode array but, instead of a train, employed an optogenetic ramp, increasing monotonically over 1 s, to activate the Layer 1 glutamatergic axons. This pattern of excitation generated a very large increase in γ-band oscillations, in every brain slice, in the medially adjacent cortical territory, which was generally maximal in Layer 2/3, as previously reported (Adesnik and Scanziani, 2010). We performed frequency domain analyses (MATLAB, pwelch.m) of the induced rhythm, relative to an equivalent duration epoch immediately before the onset of the optogenetic ramp. Although we observed considerable slice-to-slice variability, there was a marked tendency for the slices prepared at ZT5 to show higher-frequency oscillations (Fig. 7; Table 5), with highly significant differences in the median and peak frequencies and both the lower and upper limits of the half-width frequency. In summary, we found marked time-of-day differences in network assays of neocortical inhibition.

**Table 5.** Day/night differences in oscillations induced by optogenetic ramp

	ZT5 (*n* = 17) mean ± SEM	ZT17 (*n* = 16) mean ± SEM	*p* value
Peak power (µV^2^/s)	13.7 ± 4.8	8.4 ± 4.9	0.324 ns
Median frequency (Hz)	88.1 ± 6.8	65.6 ± 4.7	0.011*
Peak frequency (Hz)	85.8 ± 7.8	64.1 ± 8.0	0.036*
Half width (Hz)	46.7 ± 7.6	39.3 ± 7.8	0.445 ns
HW lower limit (Hz)	60.2 ± 7.8	36.7 ± 8.1	0.017*
HW upper limit (Hz)	106.9 ± 9.0	75.9 ± 9.2	0.008*

All distributions were normal; comparisons made using Student's *t* tests (*significant. Ns, not significant).

## Discussion

Electrochemical gradients across the cell membrane fundamentally dictate neuronal behavior. For this reason, the prevailing view is that neuronal ion content is tightly constrained, but several recent studies (Ding et al., 2016; Alfonsa et al., 2023; Pracucci et al., 2023), extended by our findings presented here, are now challenging this view. Previously, we reported an unexpectedly large physiological modulation of [Cl^−^]_in_ in pyramidal cells between day and night, and now we show that [Cl^−^]_in_ also varies over the same time period in PV interneurons but in the opposite direction (Fig. 4). This time-shifted modulation of [Cl^−^]_in_ in PV interneurons rides on top of a higher baseline mean [Cl^−^]_in_ in this population, relative to that in pyramidal cells. Similarly in organotypic cultures of the mouse hippocampus, the baseline [Cl^−^]_in_ in PV interneurons was observed to be higher than in pyramidal cells, although the daily modulation could not be studied in that preparation (Calin et al., 2023).

Interestingly, GABAergic neurons in the suprachiasmatic nucleus also show [Cl^−^]_in_ dynamics (Wagner et al., 1997) similar to what we report here in cortical PV interneurons, but clearly this is not a pattern common to all GABAergic neurons, because SST interneurons show minimal [Cl^−^]_in_ modulation, and if anything, they conform more toward the pyramidal pattern (Fig. 4).

It is well established that intense GABAergic drive favors Cl^−^ influx and may transiently overwhelm Cl^−^ efflux, leading to chloride-loading of neurons (Thompson and Gahwiler, 1989). However, an activity-dependent explanation for the large daily modulation of [Cl^−^]_in_, in different cell classes is difficult to reconcile with the recent data. First, the three cell classes we have studied (PV, SST, and pyramidal cells) sample heavily from the same presynaptic population (Markram et al., 2015; Gal et al., 2017), with their primary excitatory drive coming from other supragranular pyramidal cells and their primary inhibitory drive from local SST and PV interneurons. While the presynaptic drives are certainly not identical, the differences appear to represent the minority of inputs, meaning that any activity-dependent ionic redistribution would be expected to be correlated; instead, we found that PV interneurons and pyramidal cells show opposite phase modulation of [Cl^−^]_in_. We also observed differences between the two interneuronal classes. Second, brain slices prepared at different times of day show different levels of [Cl^−^]_in_ in pyramidal cells even in the presence of tetrodotoxin, which blocks all neuronal firing (Alfonsa et al., 2023). If the variation in [Cl^−^]_in_ arises from differences in activity levels in vivo, through the day, then this should be negligible in this preparation. Interestingly, we report here that brain slices prepared at different times of day show pronounced differences to stimulation, in addition to previous reports showing differences in the number of GABAergic receptors (Bridi et al., 2020) and how easily LTP could be induced (Chaudhury et al., 2005; Nakatsuka and Natsume, 2014; Alfonsa et al., 2025). The variation in the network responses to stimulation are consistent with the cell-class–specific changes we find in *E*_GABA_ (Fig. 5) and also with our previous analyses of visually induced oscillations in the primary visual cortex of mice, in vivo, which showed a similar pattern of day-to-night modulation (Pracucci et al., 2023).

Intracellular [Cl^−^] may also be influenced by Gibbs–Donnan effects imposed by changes in osmotic tension either at the extracellular (Glykys et al., 2014; Rahmati et al., 2021) or intracellular compartment (Stangherlin et al., 2021). This latter study, although it focuses entirely on non-neuronal cells, is of great interest because it suggests a means whereby cell classes might achieve different levels of [Cl^−^]_in_ through cell-class–specific modulation of their cytosolic milieu by, for instance, differential sensitivity to mTOR regulation.

We also found a marked difference in pH_in_ in the three classes of neurons, although, notably, pH_in_ appears stable over the course of the day in all of them. The different pH_in_ may be another cause of functional divergence between these cell classes, arising through the multiple effects of pH on various membrane channels, transporters, and pumps [reviewed in Ruffin et al. (2014)]; however, the net effect is clearly complex and may be difficult to predict without performing direct experimental manipulation of pH. One pertinent example is that GABAergic receptors are permeable to bicarbonate ions $\mathrm{HC}O_{3}^{-}$, making pH_in_ an important secondary determinant of *E*_GABA_. The more alkaline intracellular state in PV interneurons means that they have both raised [Cl^−^]_in_ and raised $[\mathrm{HC}O_{3}^{-}{]}_{\mathrm{in}}$, mutually reinforcing the positive shift in *E*_GABA_. How far this effect is offset by shunting inhibition or the action of GABA_B_ receptor-mediated inhibition or other pH-dependent changes in excitability remains to be determined.

In summary, there are several important principles arising from these various studies. First, one must consider the time of day when experiments are performed, even when animals are anesthetized, or when using in vitro preparations, since the time-of-day effects do persist. Second, there are marked cell-class–specific differences in the physiological modulation of [Cl^−^]_in_, in the mouse neocortex, through the day. To date, we have only examined a subset of all the cell classes present in the cortex and only in a single cortical location. Given the unexpectedly large size of the ionic redistribution, the existence of phase shifts in [Cl^−^]_in_ modulation between cell classes, the evident impact on functional and pathological network states and the potential for manipulating these states, we can recognize the need for further research in this area. There are currently great efforts being made to catalog the anatomical microstructure of brains (Oh et al., 2014; Kasthuri et al., 2015; Markram et al., 2015; Gal et al., 2017; Abbott et al., 2020), with the expectation that these will illuminate how activity spreads through neuronal networks. In practice, however, the flow of activity may not be predictable from such models, due to physiological factors that are cryptic (Randi et al., 2023). Indeed, the daily modulation of ionic redistribution that we have described here may deliver remarkable changes in the performance of neuronal networks without any change in the anatomical microcircuitry.

## Synthesis

Reviewing Editor: Jean Christophe Poncer, INSERM Decisions are customarily a result of the Reviewing Editor and the peer reviewers coming together and discussing their recommendations until a consensus is reached. When revisions are invited, a fact-based synthesis statement explaining their decision and outlining what is needed to prepare a revision will be listed below. The following reviewer(s) agreed to reveal their identity: Xin Tang, Jessica Pressey. Note: If this manuscript was transferred from JNeurosci and a decision was made to accept the manuscript without peer review, a synthesis may not be available.

Both reviewers emphasized the importance and quality of your work, and they agreed that your conclusions are well supported by the experimental data.

However, given the complexity of the effects described in your manuscript and in previous studies in this field, the reviewers and I strongly encourage you to provide a graphical summary or diagram illustrating how cell-type-specific circadian regulation of Cl⁻ levels may contribute to network activity.

## Author Response

We would like to thank the reviewers and editors for facilitating this publication, both through their comments and suggestions, and also by recognizing the difficulties we faced to address suggestions made in previous reviewing rounds. We were very excited by our data, and that results that will be of interest to the neuroscience community. We are very pleased that these will be published without further delay.

We are grateful also for the suggestion to include a schematic to explain the network effects of altered [Cl-]in, which we provide as Figure 5. This was an excellent suggestion and it will undoubtedly help the reader follow our arguments. We have corrected the figure referencing throughout the manuscript, and also addressed the other minor comments, and corrected a handful of typographic errors that we spotted. Our responses to the critiques are below, interleaved with the reviewers' comments.

Synthesis Statement for Author:

Both reviewers emphasized the importance and quality of your work, and they agreed that your conclusions are well supported by the experimental data.

However, given the complexity of the effects described in your manuscript and in previous studies in this field, the reviewers and I strongly encourage you to provide a graphical summary or diagram illustrating how cell-type-specific circadian regulation of Cl⁻ levels may contribute to network activity.

Reviewer 1 This manuscript builds upon the findings of Pracucci et al., 2023, who characterized circadian fluctuations in intracellular chloride concentration ([Cl⁻]ᵢₙ) in subgranular cortical pyramidal neurons using LSSM-ClopHensor imaging combined with electrophysiological recordings. Here, the authors extend this work by applying a similar imaging strategy to two major cortical interneuron subtypes: parvalbumin (PV)- and somatostatin (SST)-expressing interneurons. These results reveal that circadian [Cl⁻]ᵢₙ dynamics in PV interneurons differ from the patterns previously observed in pyramidal neurons, while SST interneurons show a non-significant circadian fluctuation in [Cl-]in, thus providing new insights that will be of particular interest to researchers studying inhibitory circuit regulation.

The authors performed highly technical imaging experiments using the chloride- and pH-sensitive biosensor ClopHensor, as well as multielectrode probe recordings combined with channelrhodopsin-mediated activation of pyramidal neurons to investigate the roles of PV- and SST-expressing interneurons. They have also provided a clearer explanation of the possible mechanisms underlying their findings. While a complete model of the oscillation remains to be established, the authors have acknowledged this limitation and added relevant references, leaving open the possibility of PV involvement.

The authors have satisfactorily addressed the original concerns raised by Reviewers 1 and 2. I have one minor edit noted below, but otherwise consider the submitted revisions sufficient. Although both reviewers emphasized the need for additional electrophysiological recordings or further ClopHensor imaging to fully characterize daytime-dependent changes in [Cl⁻]ᵢₙ, the authors have provided a clear explanation for why these experiments are not currently feasible. Taken together with the clarifications and revisions made to the manuscript, I believe the current version is suitable for publication.

Minor comments Lines 367-369: this sentence is too dense and should be clarified. "These changes are consistent with our prediction..." We have changed the text here, and provide a more extended explanation, expanding it over several, shorter sentences. In tandem with the new schematic figure, we hope that this is easier to follow. This is now on line 365 of the tracked manuscript (line 396 of the 'clean' version).

Reviewer 2 The manuscript reports novel observations on the differential chloride dynamics of SST and PV interneurons across the 24-hour cycle. This focus on interneuron subtypes, rather than pyramidal cells as in prior work, provides important new insight into the circuit basis of day-night physiological changes.

The data are solid, and the authors have clearly addressed the earlier reviewers' comments by strengthening their explanations and improving the writing.

The observations and measurements in Figures 1-3 are clear and convincing. In Figures 4-5, the authors extend their findings using circuit physiology, including feedforward inhibition and optogenetically driven oscillations, to establish the physiological relevance of the chloride changes observed between ZT5 and ZT17. To further strengthen the manuscript, it is strongly recommended that the authors include one or more schematic diagrams illustrating how day/night differences in Cl⁻ levels across cell types may contribute to the observed circuit phenomena. For instance, given that SST interneurons provide input to PV cells, would a decreased Cl⁻ load in SST neurons lead to increased activity and thereby enhanced GABAergic input onto PV cells? We have included a new figure (Fig 5), with multiple panels, to illustrate the predicted effects of the altered [Cl-]in on network activity.

Minor point:

In Figure 2C and D, the reported mouse genotype and immunostaining labels do not match and should be corrected.

Corrected.
